# Neuron navigator 2 overexpression indicates poor prognosis of colorectal cancer and promotes invasion through the SSH1L/cofilin-1 pathway

**DOI:** 10.1186/s13046-015-0237-3

**Published:** 2015-10-09

**Authors:** Fengbo Tan, Hong Zhu, Yiming Tao, Nanhui Yu, Qian Pei, Heli Liu, Yuan Zhou, Haifan Xu, Xiangping Song, Yuqiang Li, Zhongyi Zhou, Xiao He, Xingwen Zhang, Haiping Pei

**Affiliations:** Department of Gastrointestinal Surgery, Xiangya Hospital, Central South University, 87 Xiangya Road, Changsha, Hunan 410008 P.R. China; Department of Oncology, Xiangya Hospital, Central South University, Changsha, China; Department of General Surgery, Xiangya Hospital, Central South University, Changsha, China; Department of Mammary and Thyroid, The First Affiliated Hospital of South China University, Hengyang, China; Department of Mammary, The Hunan Cancer Hospital, Changsha, China; Department of Emergency, The Hunan Provincial People’s Hospital, Changsha, China

**Keywords:** NAV2, Colorectal cancer, Prognosis, SSH1L/cofilin-1 pathway, Tumor budding

## Abstract

**Background:**

Neuron navigator 2 (NAV2) encodes a member of the neuron navigator gene family, which plays a role in tumorigenesis and cell migration. However, the prognostic value of NAV2 expression in colorectal cancer (CRC) patients and the potential pathway through which NAV2 promotes migration and invasion in CRC cell lines is poorly understood.

**Methods:**

The expression level of NAV2 was detected in CRC tissues from two different CRC cohorts by immunohistochemistry, qRT-PCR and Western blotting; the correlation between NAV2 expression and clinicopathological characters was analyzed, and the prognostic value of NAV2 expression was analyzed using a Cox regression model. CRC cell lines with NAV2 knocked out were used to validate the function and potential pathway used by NAV2 to promote CRC cell migration and invasion.

**Results:**

The results showed that NAV2 was overexpressed in CRC tissues, and it was closely correlated with depth of invasion, and lymph and distant metastasis. Multivariate analysis indicated that high NAV2 expression was a poor prognostic indicator of recurrence-free survival and overall survival in CRC patients. Furthermore, Cox regression analysis revealed that high NAV2 expression integrated with high tumor budding grade was a powerful independent predictive factor of CRC clinical outcome. In vitro and in vivo assays demonstrated that knockdown of NAV2 led to reduced migration and invasion of cancer cells, and the process involved the regulation of F-actin polymerization through the SSH1L/cofilin-1 pathway.

**Conclusion:**

Based on these findings, NAV2 could serve as both a prognostic biomarker and a potential therapeutic target for patients with NAV2-positive CRC.

**Electronic supplementary material:**

The online version of this article (doi:10.1186/s13046-015-0237-3) contains supplementary material, which is available to authorized users.

## Background

Colorectal cancer (CRC) is a leading cause of cancer mortality worldwide [1]. Many improvements have been made in screening, detection and adjuvant therapy for CRC in recent years [2, 3]. However, the long-term survival associated with this malignant disease is not satisfactory due to tumor recurrence and metastasis [4]. To improve the prognosis of CRC patients, there is a great need to identify efficient new targets for effective disease management.

Neuron navigator 2 (NAV2) is one of three members of the neuron navigator family [5]. NAV2 protein contains various functional domains, including a calponin homology (CH) domain, four coiled-coil (CC) domains, a cytoskeletal interacting domain (CSID), and an AAA-domain [6]. The functional domains were shown to be involved in a variety of cellular processes, including protein degradation, signal transduction, regulation of gene expression, membrane fusion, microtubule dynamics and cell migration. It has been proven that NAV2 affects cell migration via adjusting microtubule dynamics [7, 8], and microtubule dynamics induces basement membrane breakdown, actin stress fiber formation, metal matrix protease secretion [9]. NAV2 also regulates the cytoskeleton, which affects cell-cell and cell-matrix adhesion, facilitating tumor cell invasion and metastasis [10].

The actin cytoskeleton plays a central role in cell migration by mediating processes such as cell-substrate adhesion, protrusion, phagocytosis and cytokinesis [11]. Cofilin is an essential regulator of actin dynamics, and researchers have revealed that phosphorylation and inactivation of cofilin-1 was capable of blocking actin polymerization and metastasis in cancers [12, 13]. Cofilin is inactivated by phosphorylation at Ser-3 by LIM kinase and reactivated by dephosphorylation by the cofilin-phosphatase, Slingshot (SSH) [13, 14]. Meanwhile, the NAV2 homolog, Sickie, regulates actin dynamics via the (SSH)-cofilin pathway [15]. NAV2 was deemed to be one of target genes of APC/Wnt/β-catenin, which is a signaling pathway that plays an essential role in tumor progression according to studies using the CRC cell line SW480 [16]. Moreover, NAV2 was reported to be highly expressed in neuroblastoma cells and uterine endometrial stromal sarcoma [17, 18], as well as in a small group of CRC patients [16].

In sum, we can infer that NAV2 is overexpressed in CRC, and it promotes invasion and metastasis via its impact on actin or microtubule dynamics and its involvement in the SSH1L/cofilin-1 pathway. The objectives of this study were to investigate the mRNA and protein expression levels of NAV2 in CRC, the role of NAV2 in CRC cell migration and invasion, the prognostic value of NAV2 expression in CRC patients, and the potential pathways by which NAV2 exerts its influence on actin dynamics.

## Materials and methods

### Clinical specimens and follow-up

Tumor and paired normal mucosa tissues were removed by surgery from 138 CRC patients (cohort 1) at the XiangYa Hospital (Changsha, China) between January 2007 and December 2008. The samples were then embedded in paraffin. Follow-up of patients in cohort 1 was terminated on March 30, 2015. Recurrence-free survival (RFS) was defined as time to any event, irrespective of cause, except for any second primary cancers. Recurrence of or death from the same cancer and all treatment-related deaths or deaths from other causes are events. Second primary same cancers and other primary cancers are ignored, and loss to follow-up is censored. Overall survival (OS) was defined as time to death, irrespective of cause. Local recurrence, distant metastases, second primary colorectal cancers, and second other primary cancers are ignored. Loss to follow-up is censored [19]. Fresh cancerous colorectal and paired normal mucosa tissues from 76 patients (cohort 2) were procured from the Department of General Surgery of XiangYa Hospital of Central South University between December 2013 and August 2014. Paired tissues were tested by real-time qRT-PCR, and 16 paired tissues were selected randomly for Western blotting analysis. Primary tumor and paired metastatic tumor tissues (lymph node, peritoneal metastasis or liver metastasis) from 16 patients were selected for Western blotting analysis. All procedures were in compliance with the ethical guidelines of the XiangYa Hospital. The normal mucosa was excised 5 cm away from the tumor and was confirmed by pathologists for absence of tumor cells. Tumor stage was classified according to the 7th edition of the AJCC TNM staging system for CRC.

### Estimation of tumor budding and immunohistochemical staining

Hematoxylin-eosin (H & E) staining was performed on 138 CRC samples. The degree of tumor budding was classified as low-grade or high-grade, corresponding to 0–9 and ≥10 in one 20× field, respectively [20]. The protocol for immunohistochemical staining was performed according to a previous report [17]. The tissue slides were deparaffinized, and antigen retrieval was performed by immersing the slides into boiling EDTA-Tris buffer (pH 8.2) for 4 min. After incubation with 3 % H_2_O_2_for 15 min, slides were incubated with primary antibody (Rabbit polyclonal to NAV2, Abcam, ab101790, 1:1000) overnight (4 °C), followed by incubated with secondary antibody for 30 min at room temperature. DAB (3,3-diaminobenzidine) was used to visualize positive immune reaction. Nuclei were counterstained with hematoxylin [21]. Briefly, the sections were scored using a foutier scale according to percentage of positive cells and staining intensity: (−) or 0, tissue specimens without staining (0–10 %); (+) or 1, tissue specimens with weak staining (10–25 %); (++) or 2, tissue specimens with moderate staining (25–50 %) and (+++) or 3, tissue specimens with strong staining (>50 %). (−) and (+) were defined as low expression, and (++) and (+++) were defined as high expression (or over-expression) [22]. The result of staining was evaluated independently by two board-certified clinical pathologists blinded to the clinical parameters. Any discrepancy between the two evaluators was resolved by re-evaluation and careful discussion until agreement was reached.

### Cell culture and antibodies

Cell lines NCM460, HRT-18, Caco2, SW620, SW480, HCT-116, LoVo, A549 and HepG2 were purchased from the Cell Centre of Xiang Ya Medicine College of the Central South University (Changsha, China); AGS was obtained from the Xiang ya Central Experiment Laboratory, Central South University. HT-29 and RKO Cells were a gift from Doctor Duan Q (from the Xiang Ya Hospital of Central South University). All cells were cultured in 1640 (Biological Industries, State of Israel). All media were supplemented with 10 % fetal bovine serum (Biological Industries, State of Israel). Cells were cultured at 37 °C in humidified air with 5 % CO_2_. Primary antibodies: (NAV2, SSHIL, cofilin1, p-cofilin1, Abcam; β-actin, CST; p-SSHIL, ECM Biosciences).

### RNA extraction and real-time qRT-PCR

Total RNA was extracted from cells and tissues using TRIzol Reagent (TAKARA, JAPAN), and equal amounts of RNA were used for real-time qRT-PCR analysis (TAKARA, JAPAN) according to the manufacturer’s instructions. β-actin was used as an internal control. Below are the specific primers used: NAV2, sense, 5’- GAGGGACGGGAGTTGACAGA- 3’ and antisense 5’- CAGTTGAGCAGCCCATTGAA-3’; β-actin, sense, 5’-GCACCACACCTTCTACAATGAGC - 3’ and antisense 5’- GGATAGCACAGCCTGGATAGCAAC-3’. The 2^-ΔΔCT^ method was used to calculate the relative abundance of RNA for each gene compared with β-actin expression. Each reaction was performed in triplicate.

### Western blotting

Tissue samples and colonic cancer cells were homogenized and lysed in strong RIPA buffer supplemented with proteinase inhibitors. Equal amounts of proteins were loaded and separated on a 6 % SDS-PAGE (sodium dodecyl sulfate-polyacrylamide gel electrophoresis) gel. Following electrophoresis, the proteins were transferred to a PVDF membrane (Millipore, Billerica, MA), the membrane was blocked in 5 % (w/v) non-fat milk and incubated with the primary antibodies overnight, and then secondary antibody (1:2000 dilution, CST, USA) for 1 h. Bands were visualized and quantitated using the ECL Advance Detection System (Millipore, Billerica, MA). The dilution of primary antibody (NAV2, 1:2000; SSHIL, 1:5000; cofilin1, 1:1000; p-cofilin1, 1:1000; p-SSHIL, 1:1000; β-actin, 1:1000).

### shRNA transfection

NAV2 shRNA and the matching negative control shRNA were purchased from the GENECHEM Company (Shang Hai, China). The shRNAs were transfected into HCT-116 and LoVo according to the instructions. Cells were seeded in six-well plates at a density of 1 × 10^5^ cells/well. The transfection reagent and negative control shRNA -transfected cells were used as control.

### Immunofluorescence

Cells cultured in 24-well chamber slides were washed twice with cold phosphate-buffered saline (PBS), fixed with 4 % paraformaldehyde for 10 min, permeabilized with 0.1 % Triton ×-100 for 5 min, blocked with 5 % BSA, and incubated with the indicated antibodies for F-actin at 4 °C overnight (Phalloidin-TRITC, Sigma-Aldrich. 1:100). The cells were then stained with DAPI (4,6-diamidino-2-phenylindole) to visualize the nuclei, and the images were acquired with a fluorescence microscope.

### In vitro motility and invasion

For the scratch wound healing assay, cells were grown to confluence in a 6-well plate and were wounded using a 20 μl sterile pipette tip. Wound healing within the scraped line was documented daily over 36 h, and repeated at least three times in duplicate. The cells’ invasive ability was measured in 24-well transwell chambers (Corning, USA). 10 % FBS was added to the bottom chamber as a chemo-attractant. For the invasion assay, Matrigel (1:4, BD Biosciences, USA) was added to the transwell membrane chambers and incubated at 37 °C for 4 h. Cells (1 × 10^5^ cells per well) were seeded and incubated for 36 h before analysis. Non-migrating cells on the upper side of the membrane were removed, and the cells that migrated to the lower chamber were fixed with 4 % paraformaldehyde and stained with Wright-Giemsa to assess invasion. The number of invading cells was determined for five randomly selected fields under a microscope. Each experiment was performed on at least three occasions in duplicate.

### In vivo tumor metastasis

HCT-116 and LoVo cells transfected with shNAV2 and empty vector were suspended in 1640, then injected into the spleen of athymic nude mice (2 × 10^6^ cells/mouse, 5 mice per group). The spleen was resected 30 min after injection. Tumor volume was calculated according to the following formula: length × (width)^2^/2. Mice were sacrificed at day 42. All animals received humane care according to the Institutional Animal Care and Treatment Committee of Central South University.

### Statistical analysis

Statistical analysis of the differences in the expression levels of NAV2 mRNA in paired tissues were analyzed by the paired t-test. Correlation between NAV2 expression and clinicopathological factors was estimated by the chi-square test. RFS and OS curves were plotted according to the Kaplan-Meier method; A Cox regression model was used to perform multivariate analysis, parameters of significance in univariate analysis were included in the multivariate analysis. The statistical between ≥2 groups was analyzed by Student’s t-test or one-way analysis of variance. The correlation between NAV2 expression levels and tumor budding grades were determined Spearman’s correlation analysis. Statistical *P*-values < 0.05 were considered significant. The statistical software programs used were Prism 5 (Graph Pad Software Inc., La Jolla, CA, USA) and SPSS 13.0 for Windows (SPSS, Chicago, IL).

## Results

### NAV2 was overexpressed in CRC tissue and associated with the prognostic outcome of CRC patients

**Fig. 1:**
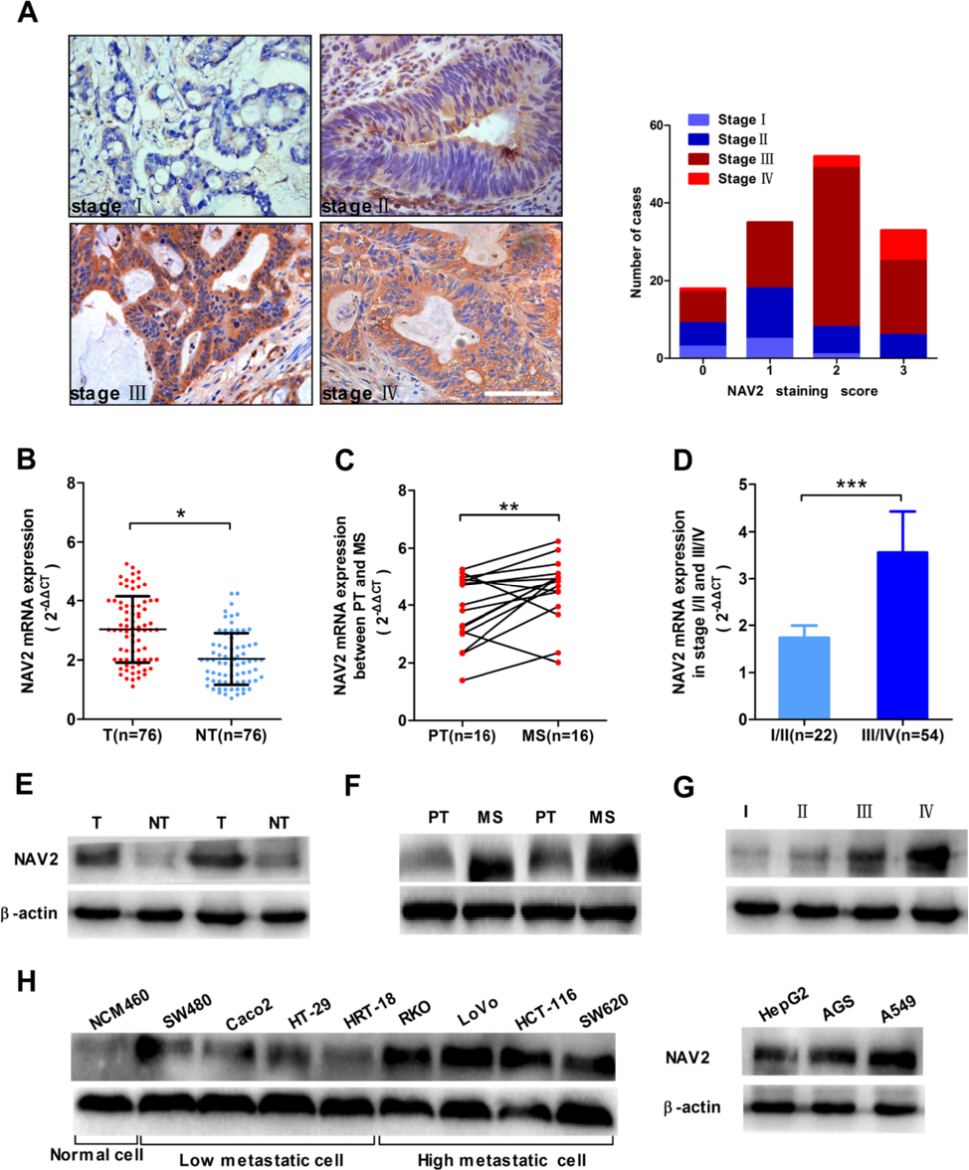
Overexpression of NAV2 in CRC and was associated with more aggressive tumor stage: **a** Immunohistochemical staining of NAV2 with increased score ranged from stageIto IV in CRC patients from cohort 1. Magnification: ×400; scale bar, 50 μm. NAV2 mRNA level was detected by qRT-PCR in CRC patients from cohort 2 (B-D): **b**, Tumor tissues (T) were higher than paired normal tissues (NT) (*P < 0.0001); **c**, Metastatic sites (MS) were higher than primary tumors (PT) (**P = 0.0142); **d**, Stage III/IV with higher expression level than satgeI/II (***P < 0.0001). Expression levels were normalized to those of β-actin (2^-ΔΔCT^). Western blotting was used to analysis NAV2 protein expression level in CRC patients from cohort 2 (**e**, **f**, **g**), the dates were shown in the Additional file 3: Figure S1. **h** NAV2 protein expression level in various metastatic potency CRC cells and other cell lines were tested by Western blotting, NAV2 was high expression in high metastatic potency cells, experiments have been repeated three times

**Fig. 2:**
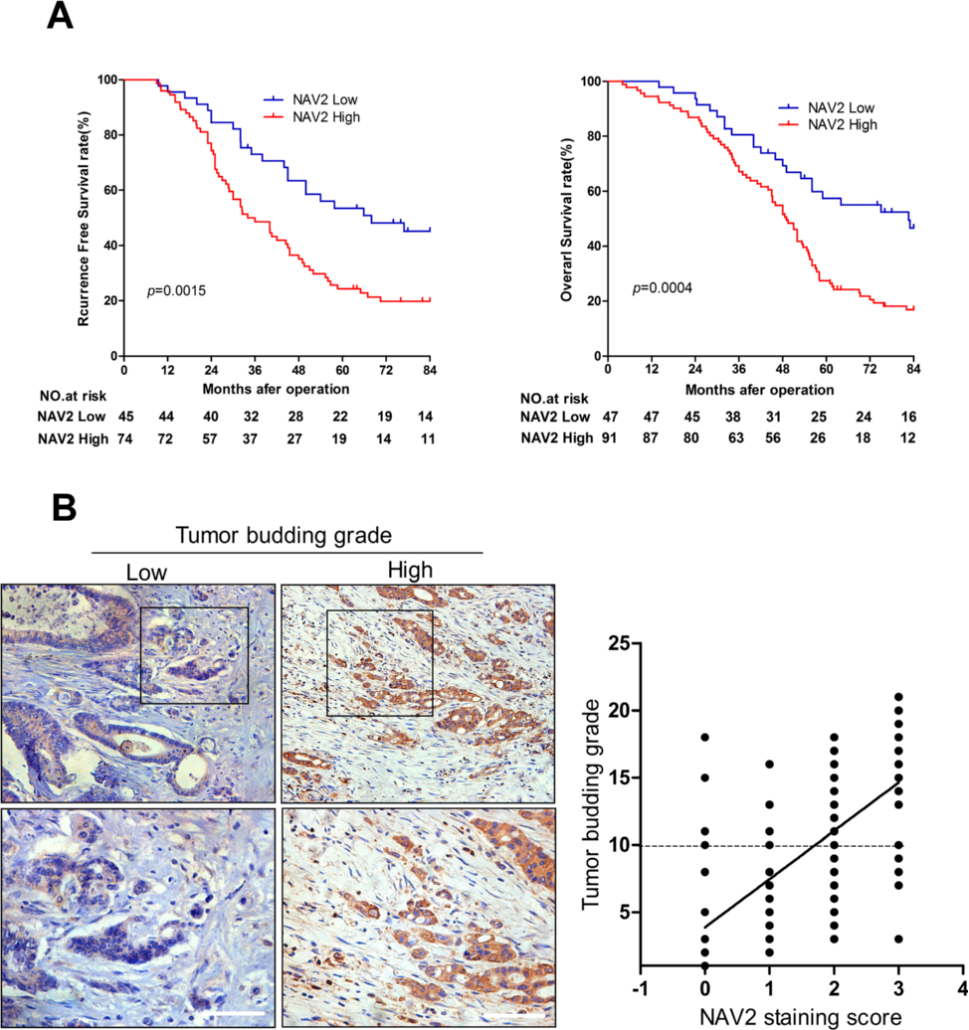
Kaplan-Meier plots with log rank test of Recurrence-free survival and Overall survival. **a** NAV2-positive tumors had a significantly lower rate Recurrence-free survival (*p* = 0.0015) and Overall survival (*p* = 0.0004) than those with NAV2-negative tumors. **b** A significant increase tumor budding grade ahead of the invasive front was observed with high NAV2 expression compared to low NAV2 expression. Magnification:×400; scale bar, 50 μm. Tumor budding grade ahead of the invasive front was positive correlation with expression level of NAV2 (*r* = 0.621, *p* < 0.0001)

We investigated the relationship between NAV2 protein or mRNA expression and clinicopathologic features in cohort 1 or cohort 2 (Table 1; Additional file 1: Table S1). NAV2 mRNA (Fig. 1b, paired t-test, *p* < 0.0001) and protein (Fig. 1e, paired t-test, *p* < 0.0001; Additional file 2: Table S2) expression in tumor tissues were higher than that in paired normal tissues. NAV2 expression was significantly correlated with TNM stages in cohort 1 (Table 1, chi-square test, *p* < 0.0001) and cohort 2 (Fig. 1d, Student’s t-test *p* < 0.0001), depth of invasion, lymph node status and distant metastasis. However, no significant correlation was found between NAV2 expression and other clinicopathologic variables studied such as age, gender, tumor location, tumor differentiation, adjuvant therapy and CEA. In addition, we found that NAV2 mRNA (Fig. 1c, paired t-test, *p* = 0.0142) and protein (Fig. 1f*,* paired t-test, *p* = 0.0043) expression in metastatic tumor tissue were higher than in paired primary tumor tissue. Based on these findings, we can conclude that high expression of NAV2 was associated with more aggressive biological behavior. To assess the predictive role of NAV2 for prognosis, Kaplan-Meier curves with a log-rank test for RFS and OS were undertaken in cohort 1 patients. There was a significant different RFS between NAV2-positive and NAV2-negative groups after the surgery (Fig. 2a, log-rank test, *p* = 0.0015). NAV2-positive tumors had a significantly lower OS rate than those with NAV2-negative tumors (Fig. 2a, log-rank test, *p* = 0.0004).

**Table 1:** Correlation between NAV2 protein expression and clinicopathological features of 138 CRC patients (cohort 1)

Clinicopathological features			NAV2 expression		
		n	Low (*n* = 47)	High (*n* = 91)	*P* value
Age (years)	<65	67	25	42	0.433
	≥65	71	22	49	
Gender	Male	80	30	50	0.316
	Female	58	17	41	
Tumor location	Left colon	45	16	29	0.408
	Right colon	45	12	33	
	Rectum	48	19	29	
Tumor differentiation	Well-Moderate	72	24	48	0.851
	Poor	66	23	43	
TNM stage	I-II	37	27	10	< 0.0001
	III-IV	101	20	81	
Depth of invasion	T1-T2	43	27	16	< 0.001
	T3-T4	95	20	75	
Lymph node metastasis	N0	39	23	16	0.0001
	N1-N2	99	24	75	
Distant metastasis	M0	119	45	74	0.020
	M1	19	2	17	
CEA	<5 ng/ml	85	31	54	0.449
	≥5 ng/ml	53	16	37	
Adjuvant therapy	YES	94	35	59	0.250
	NO	44	12	32	

*P* < 0.05 was considered statistically significant. The *P* values of chi-square test

The univariate and multivariate analysis for association of prognostic markers of CRC with RFS and OS are summarized in Table 2. Depth of invasion, lymph node status, distant metastasis and NAV2 expression were found to be prognostic factors for RFS and OS by univariate analysis (RFS, *HR* = 1.616, 95 % *CI*: 1.094–3.205, *p* = 0.014; OS, *HR* = 1.364, 95 % *CI*: 1.058–2.245, *p* = 0.021). Multivariate analysis revealed that lymph node status, distant metastasis and NAV2 expression were independent prognostic factors that could affect RFS and OS of CRC patients (RFS, *HR* = 1.785, 95 % *CI*: 1.108–2.528, *p* = 0.010; OS, *HR* = 1.564, 95 % *CI*: 1.108–2.376, *p* = 0.012).

**Table 2:** Univariate and multivariate analysis of 138 CRC patients for RFS and OS (cohort 1)

Variables	RFS				OS			
	Univariate analysis		Multivariate analysis		Univariate analysis		Multivariate analysis	
	HR (95 % CI)	*P* value	HR (95 % CI)	*P* value	HR (95 % CI)	*P* value	HR (95 % CI)	*P* value
Age, years (≥65 vs. <65)	1.043 (0.756–1.132)	0.215	NA		1.038 (0.694–1.367)	0.325	NA	
Gender (male vs. female)	1.162 (0.964–1.376)	0.078	NA		1.132 (0.924–1.764)	0.086	NA	
Tumor location (left colon vs. right colon vs. rectum)	1.055 (0.821–1.248)	0.133	NA		1.127 (0.885–1.526)	0.134	NA	
Differentiation (poor vs. well/ moderate)	1.204 (0.964–1.483)	0.061	NA		1.267 (0.963–1.837)	0.065	NA	
TNM stage (III/IVvs.I/II)	1.436 (1.078–2.032)	0.020	1.321 (1.054–2.421)	0.025	1.512 (1.093–2.162)	0.016	1.897 (1.094–3.528)	0.013
Depth of invasion (T3/T4 vs. T1/T2)	1.267 (1.032–1.628)	0.037	1.072 (0.967–1.468)	NS	1.284 (1.083–1.764)	0.028	1.173 (0.957–1.528)	NS
LN metastasis (N1N2 vs. N0)	1.452 (1.082–1.936)	0.019	1.281 (1.032–1.846)	0.035	1.301 (1.075–1.964)	0.033	1.513 (1.128–3.127)	0.013
Distant metastasis (M1 vs. M0)	1.852 (1.137–2.814)	0.008	1.536 (1.108–2.162)	0.021	1.564 (1.109–2.352)	0.012	1.305 (1.106–2.642)	0.018
CEA (≥5 ng/ml vs. <5 ng/ml)	1.164 (0.925–1.637)	0.092	NA		1.173 (0.867–1.467)	0.124	NA	
Adjuvant therapy (NO vs. YES)	1.223 (0.985–1.921)	0.058	NA		1.252 (0.964–1.682)	0.072	NA	
NAV2(high vs. low)	1.616 (1.094–3.205)	0.014	1.785 (1.108–2.528)	0.010	1.364 (1.058–2.245)	0.021	1.564 (1.108–2.376)	0.012
Tumor budding (high vs. low)	1.974 (1.206–4.267)	0.005	2.021 (1.156–3.126)	0.004	1.852 (1.271–6.324)	0.006	2.114 (1.247–4.826)	0.002
^a^Combined NAV2 and Tumor budding								
II vs.I	2.021 (1.316–5.416)	0.002	2.157 (1.206–5.824)	0.003	1.936 (1.314–5.735)	0.004	2.052 (1.126–3.843)	0.003
III vs.I	4.828 (1.862–9.072)	0.000	5.126 (1.935–9.675)	0.000	4.352 (2.134–8.846)	0.000	4.723(1.925–9.506)	0.000
III vs.II	2.384 (1.267–7.321)	0.001	2.324 (1.221–6.326)	0.000	2.153 (1.426–6.875)	0.001	2.231(1.286–6.251)	0.0005

Abbreviations: *HR* hazard ratio, *CI* confidence interval, *NA* not available, *NS*, not significant

^a^I (low risk group): NAV2Low/Tumor budding Low; II (medium-risk group): NAV2High/Tumor budding Low or NAV2Low/Tumor budding

### The combination of NAV2 overexpression and high tumor budding grade served as independent prognostic markers for RFS and OS

When analyzing the levels of NAV2 expression in CRC tissues by immunohisto chemistry, we found an interesting phenomenon that high NAV2 expression was accompanied by a high tumor budding grade. The result of the analysis showed that high tumor budding grade ahead of the invasive front was positively correlated with late TNM stage (Spearman’s correlation, *r* = 0.3760, *p* < 0.0001) and high expression level of NAV2 (Fig. 2b, Spearman’s correlation, *r* = 0.6208, *p* < 0.0001). Multivariate analysis indicated that tumor budding grade was an independent prognostic factor that could affect RFS and OS of CRC patients (RFS, *HR* = 2.021, 95 % *CI*: 1.156–3.126, *p* = 0.004; OS, *HR* = 2.114, 95 % *CI*: 1.247–4.826, *p* = 0.002). Meanwhile, univariate and multivariate analysis indicated that high NAV2 expression combined with high tumor budding grade is associated with shorter RFS and OS than other combinations with a different status of the two factors (Table 2). In summary, NAV2 and tumor budding were independent prognostic factors that could affected RFS and OS of CRC patients.

### Inhibition of NAV2 reduced migration and invasion of CRC cells and tumor metastasis in vitro and vivo

**Fig. 3:**
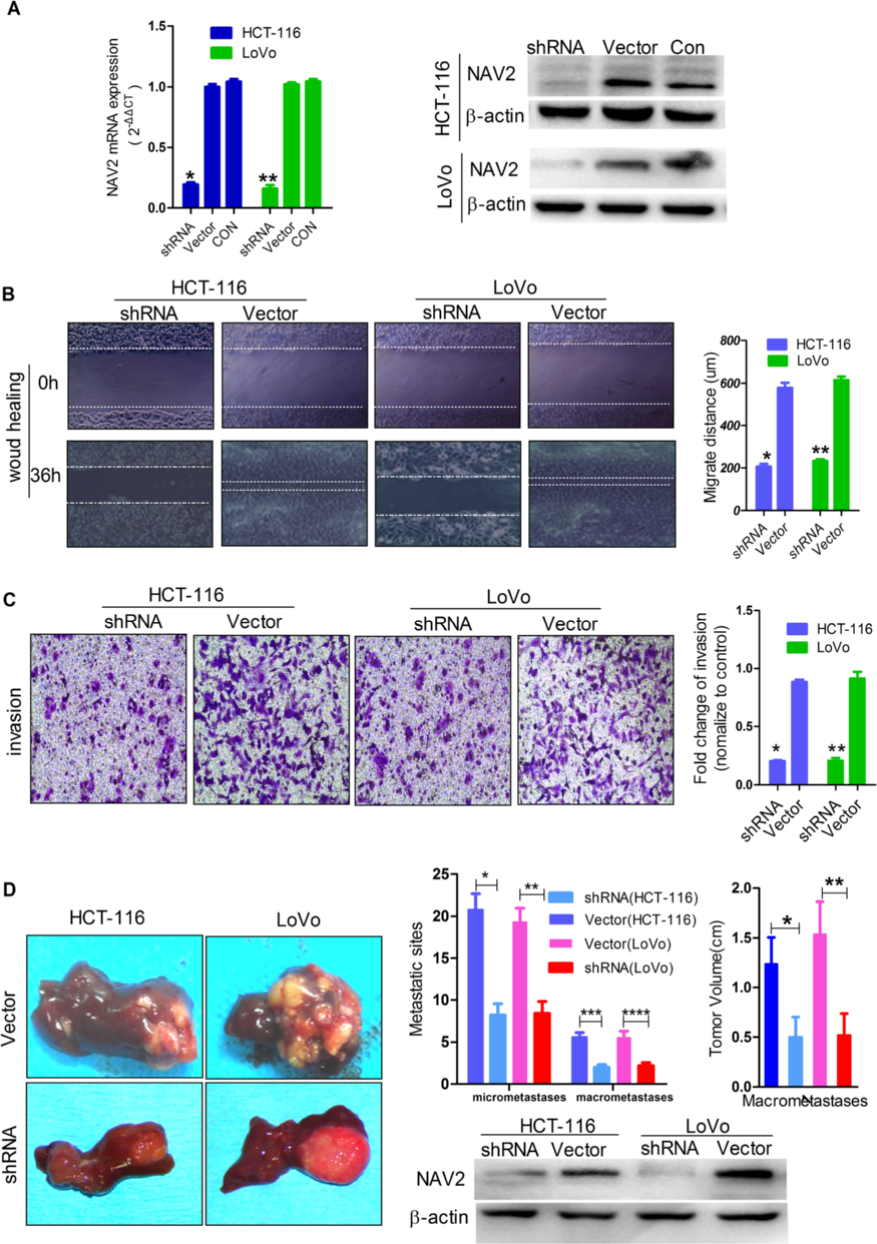
Knockdown NAV2 inhibited migration and invasion of CRC cells in vitro and vivo. **a** Inhibition of NAV2 expression level in HCT-116/ LoVo cells were testified by qRT-PCR (One-way analysis, both *P* < 0.0001) and Western blotting. Experiments have been repeated three times **b** Wound-healing assay for NAV2 shRNA HCT-116 (Student’s t-test, *P = 0.0002) and shRNA LoVo (Student’s t-test, **P < 0.0001) compared with empty vector cells. Photographs were taken at 36 h post wounding. Dashed lines indicated the original wound boundaries. Experiments have been repeated three times. **c** Cell invasion assays, results were presented as the ratios of migrated NAV2 shRNA cells relative to those of empty vector cells HCT-116 (Student’s *t*-test, *P < 0.0001) and LoVo (Student’s t-test, **P = 0.0003). Data were representative of three experiments .Original magnification, ×100. **d** Representative liver metastatic tumors were shown between shRNA cells and empty vector cells; Quantification of the liver macrometastases and micrometastases: shRNA HCT-116 and empty vector cells (Student’s t-test, ***P = 0.0003 *P = 0.0006); shRNA LoVo and empty vector cells (Student’s t-test, ****P = 0.0023,**P = 0.0008); The average tumor volume of shRNA and the empty vector cells: HCT-116, (Student’s t-test, *P = 0.0012); LoVo, (Student’s t-test, **P = 0.0004); The extent of silencing by shRNA in vivo was verified by Western blotting (*bottom righ*t)

To investigate the expression level of NAV2 in various cell lines including different metastatic potency CRC cells [23–25], NAV2 protein expression levels were tested by Western blotting (Fig. 1h). The result showed that NAV2 was more highly expressed in strong metastatic potency cell lines than weak metastatic potency cell lines, and the expression level of metastatic cell line SW460 was higher than that of primary tumor cell line SW480. The efficacy of NAV2 knockdown was confirmed by real-time PCR and Western blot analyses in HCT-116/ LoVo cell lines (Fig. 3a). In the wound healing assay, NAV2 shRNA HCT-116/LoVo cells migrated a much shorter distance into the wound area than the empty vector cells (Fig. 3b). Consistent with this observation, fewer NAV2-silenced cells invaded into the lower chamber than empty vector cells (Fig. 3c).

A liver metastatic assay was performed in nude mice to examine the in vivo metastatic potential of NAV2 shRNA HCT-116/LoVo cells and empty vector cells (Fig. 3d). As we can see from the results, the average tumor volume of NAV2 shRNA HCT-116/LoVo cells was smaller than that of the empty vector cells. The average number of both micrometastases and macrometastases in mice liver following injection of NAV2 shRNA cells was fewer than those in mice injected with the empty vector cells as determined by H & E staining. The extent of silencing by the shRNA vector in vivo was verified by Western blotting. In general, these results show that NAV2 could promote migration and invasion of CRC cells.

### Knockdown of NAV2 influenced F-actin polymerization through the SSH1L/cofilin-1 pathway

**Fig. 4:**
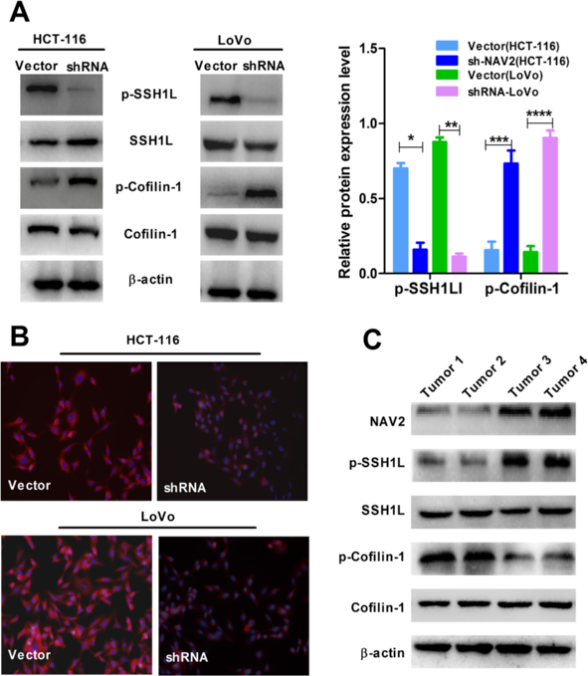
Loss of NAV2 expression decreased the phosphorylation of SSH1L but increased the phosphorylation of cofilin-1. **a** The expressions of p-SSH1L, SSH1L, p-cofilin-1 and cofilin-1 were detected by Western blotting analysis. Level of p-SSH1L and p-cofilin-1 protein in shRNA and empty vector HCT-116 (Student’s t-test, *P < 0.0001, ***P = 0.0006); Level of p-SSH1L and p-cofilin-1 protein in shRNA and empty vector LoVo cells (Student’s t-test, **P < 0.0001 ****P < 0.0001). Experiments have been repeated three times. **b** F-actin polymerization and stress fiber disassembly decreased in NAV2-depleted HCT-116/LoVo cells. Experiments have been repeated three times. **c** The expressions level of p-SSH1L, SSH1L, p-cofilin-1 and cofilin-1 in CRC tissues with different NAV2 expression level were detected by Western blotting analysis

To elucidate the potential pathway through which NAV2 promotes cell invasion, it was necessary to validate the effects of NAV2 on the downstream phosphorylation of SSH1L and cofilin-1 in CRC cells. Here, we found that knockdown of NAV2 dramatically decreased phosphorylation of SSH1L and increased phosphorylation of cofilin-1 compared with control cell lines (Fig. 4a). Cancer cell migration and invasion were associated with actin polymerization [26]. As demonstrated by the results, F-actin polymerization and stress fiber disassembly were decreased in NAV2 shRNA HCT-116/LoVo cells (Fig. 4b). The expression of p-SSH1L, SSH1L, p-cofilin-1 and cofilin-1 in CRC tissues with different NAV2 expression levels were detected by Western blotting analysis (Fig. 4c). High NAV2 expression level was accompanied by increased phosphorylation of SSH1L and decreased phosphorylation of cofilin-1 compared with low NAV2 expression level.

## Discussion

There have been varying results concerning the roles of NAVs in tumorigenesis and tumor progression [17, 27, 28]. Deletion of NAV3 was detected in colorectal adenomas, cancer tissue and cell lines, the NAV3 aberrations provided two growth advantages to a subpopulation of microsatellite stability CRC linked to inflammation and cell proliferation pathways [29]. Previous studies have highlighted that NAV2 was abundantly expressed in 16 of 20 colon cancers examined but hardly detectable in corresponding non-cancerous mucosae using semi-quantitative RT-PCR, NAV2 showed 3’ to 5’ helicase activity and exonuclease activity in vitro as an oncogene. The correlation between NAV2 expression and clinical outcomes in human CRC has not been investigated. Here, we show that NAV2 protein and mRNA are highly expressed in primary CRC, taking advantage of diverse detection methods in two different cohorts. Our results are consistent with previous findings [17, 18]. Contemporary, NAV2 high expression was closely correlated with invasion depth, and lymph node and distant metastases, suggesting that NAV2 not only plays a key role in tumorigenesis but also functions in CRC progression. Multivariate analysis indicates that NAV2 overexpression is a negative prognostic factor in CRC patients in term of the RFS and OS.

Tumor budding is defined as the presence of single tumor cells or small groups of up to five tumor cells at the invasive tumor front [30]. Previous studies have reported that tumor budding often occurs ahead of the defined invasive edge in CRC tissues [31], and the presence of budding was significantly correlated with a higher rate of metastasis and poor prognosis of patients [32, 33]. An association between dynamics of cytoskeletal regulation and tumor budding grade was demonstrated in CRC [34]. Meanwhile, AAA+ protein family members, which share homology with one of NAV2 functional protein domains, have also been shown to have increased expression in CRC tumor budding [35]. According to our findings, NAV2 was overexpressed in high tumor budding grade CRC samples, and its expression level was positively correlated with tumor budding grade. Univariate and multivariate analysis indicated that tumor budding served as an independent prognostic marker for RFS and OS in CRC patients. When evaluating NAV2 and tumor budding in combination, patients in the low risk group achieved the longest RFS and OS, whereas those in the high risk group had the shortest RFS and OS. In sum, NAV2 may promote tumor budding generation, but the specific mechanisms require further research.

CRC cells undergo a series of pathologic events during the metastatic process, including invading deep into the intestinal wall, entering into the circulatory or lymphovascular system, arresting at a remote site, proliferation and induction of angiogenesis [36]. Certainly, lymph node and distant metastasis in CRC patients is a negative prognostic factor [37]. In our study, we found that NAV2 expression in lymph node and liver metastases was much higher than in matched primary CRCs, suggesting that NAV2 might be continuously activated throughout CRC development. In an in vitro study, we confirmed that NAV2 expression level is correlated with metastatic potency in various cell lines, and depletion of endogenous NAV2 in high metastatic potency cell lines, HCT-116 and LoVo, markedly diminished their migratory and invasive abilities. Subsequently, the result of liver metastatic modeling showed that shNAV2 HCT-116 and LoVo cell lines have a decreased number of macrometastatic or micrometastatic sites compared with the empty vector group.

These data, in concert with the present findings, support a critical role of NAV2 in regulating invasion and metastasis of CRC. However, so far, the mechanism by which NAV2 promotes tumor cell invasion and metastasis remains unclear, and further studies are needed to elucidate this. Previous researchers reported that NAV2 may act as a downstream target of APC-β-catenin-TCF signaling and was related to the Myc, APC gene [16, 38]. The NAV2 gene was found to be frequently fused to the Wnt pathway TCF7L1 gene as a transcription factors involved in CRC reported by the Cancer Genome Atlas Network [39]. Researchers have observed that Sickie, which shares structural similarities with the human neuron navigator 2 (NAV2), regulated F-actin polymerization via activating the SSH/cofilin pathway. At the same time, NAV2 activated the SSH1L/cofilin-1 pathway and facilitated tumor cell migration and invasion through F-actin polymerization. We found that knockdown of NAV2 decreases the phosphorylation of SSH1L but increases the phosphorylation of cofilin-1 in CRC cells. Similar results were observed in studies of CRC tissues. The immunofluorescence results showed that loss of expression of NAV2 could regulate F-actin depolymerization [40], which plays an essential role in tumor migration and invasion [41]. Therefore, we concluded that NAV2 plays a key role in promoting CRC invasion and metastasis by regulating F-actin polymerization via the SSH1L/cofilin-1 pathway.

## Conclusions

In summary, we observed that NAV2 was overexpressed in human CRC, and this overexpression was associated with an unfavorable prognosis in CRC patients. Furthermore, our data demonstrate that NAV2 promotes CRC invasion and metastasis by regulating F-actin polymerization via the SSH1L/cofilin-1 pathway and boosting tumor budding generation in terms of pathologic morphology. These results highlight the importance of better understanding the role of NAV2 in CRC pathogenesis and suggest that NAV2 could be a potential therapeutic target. However, the generalizability of our findings is limited because this is a single-center study. Thus, larger prospective studies are needed to further validate our results, and specific mechanism need to be further explored.

## Additional files

Additional file 1: Table S1. Correlation between NAV2 mRNA expression and clinicopathological features in 76 CRC patients (cohort 2) (DOC 40 kb). Additional file 2: Table S2. The intensity of staining for NAV2 in 138 paired CRC cancer and normal mucosa samples (cohort 1) (DOC 28 kb). Additional file 3: Figure S1. NAV2 protien expression level was detected by Western blotting in tumor tissues (T) and metastatic site (MS) were higher than paired normal tissues (NT) and primary tumor (PT) (*P* value calculated by paired t-test,**P*< 0.0001; ***P*=0.0043) (DOC 56 kb).
