# Supplementary material for: Neuron navigator 2 overexpression indicates poor prognosis of colorectal cancer and promotes invasion through the SSH1L/cofilin-1 pathway
Source: J Exp Clin Cancer Res. 2015 Oct 9;34:117. doi: 10.1186/s13046-015-0237-3 (PMC4600204; doi:10.1186/s13046-015-0237-3)
Supplement: Additional file 1: Table S1. — Correlation between NAV2 mRNA expression and clinicopathological features in 76 CRC patients (cohort 2) (DOC 40 kb). [file 13046_2015_237_MOESM1_ESM.doc]

**Supplementary table 1.Correlation between NAV2 mRNA expression and clinicopathological features in 76 CRC patients (cohort 2)**

| Clinicopathological Features |  | n |  | NAV2mRNA* | ***P* value** |
| --- | --- | --- | --- | --- | --- |
| Age(years) | <65 | 38 |  | 3.08±1.20 | 0.5146 |
|  | ≥65 | 38 |  | 2.98±1.04 |  |
| Gender | Male | 32 |  | 3.21±1.11 | 0.2272 |
|  | Female | 44 |  | 2.89±1.11 |  |
| Tumor location | Left colon | 29 |  | 2.85±1.04 |  |
|  | Right colon | 30 |  | 2.98±0.94 | 0.2856 |
|  | Rectum | 17 |  | 3.41±1.43 |  |
| Tumor differentiation | Well-Moderate | 50 |  | 2.99±1.16 | 0.6636 |
|  | Poor | 26 |  | 3.10±1.05 |  |
| TNM stage | I/II | 22 |  | 1.73±0.06 | <0.0001 |
|  | III/IV | 54 |  | 3.56±0.12 |  |
| Depth of invasion | T1-T2 | 12 |  | 1.68±0.40 | <0.0001 |
|  | T3-T4 | 64 |  | 3.28±1.11 |  |
| Lymph node metastasis | N0 | 25 |  | 2.10±1.04 | 0.0266 |
|  | N1N2 | 51 |  | 3.49±0.83 |  |
| Distant metastasis | M0 | 68 |  | 2.86±0.97 | 0.0068 |
|  | M1 | 8 |  | 4.50±1.27 |  |
| CEA | <5ng/ml | 34 |  | 2.77±1.22 | 0.0583 |
|  | ≥5ng/ml | 42 |  | 3.24±0.99 |  |

a,* value =2^-△△CT^.

b, *P* < 0.05 was considered statistically significant. The *P* values of Student’s t-test .
