# Supplementary material for: Neuron navigator 2 overexpression indicates poor prognosis of colorectal cancer and promotes invasion through the SSH1L/cofilin-1 pathway
Source: J Exp Clin Cancer Res. 2015 Oct 9;34:117. doi: 10.1186/s13046-015-0237-3 (PMC4600204; doi:10.1186/s13046-015-0237-3)
Supplement: Additional file 2: Table S2. — The intensity of staining for NAV2 in 138 paired CRC cancer and normal mucosa samples (cohort 1) (DOC 28 kb). [file 13046_2015_237_MOESM2_ESM.doc]

**Supplementary table 2.The intensity of staining for NAV2 in 138 paired CRC cancer and normal mucosa samples (cohort 1)**.

|  | The expression of staining for NAV2 | | | | |  | *P* value |
| --- | --- | --- | --- | --- | --- | --- | --- |
|  | Low exppression | |  | High exppression | | χ² |
|  | ─ | + |  | ++ | +++ |  |
| Tumor(n) | 15 | 32 |  | 42 | 49 | 26.96 | <0.0001 |
| Normal mucosa(n) | 33 | 54 |  | 32 | 19 |

*P* < 0.05 was considered statistically significant. The *P* values of chi-square test.
