# Supplementary material for: Neuron navigator 2 overexpression indicates poor prognosis of colorectal cancer and promotes invasion through the SSH1L/cofilin-1 pathway
Source: J Exp Clin Cancer Res. 2015 Oct 9;34:117. doi: 10.1186/s13046-015-0237-3 (PMC4600204; doi:10.1186/s13046-015-0237-3)
Supplement: Additional file 3: Figure S1. — NAV2 protien expression level was detected by Western blotting in tumor tissues (T) and metastatic site (MS) were higher than paired normal tissues (NT) and primary tumor (PT) (P value calculated by paired t-test,*P< 0.0001; **P=0.0043) (DOC 56 kb). [file 13046_2015_237_MOESM3_ESM.doc]

**Supplementary figure 1.**NAV2 protien expression level was detected by Western blotting in tumor tissues(T) and metastatic site (MS) were higher than paired normal tissues(NT) and primary tumor (PT)(*P* value calculated by paired t-test ,**P*< 0.0001; ***P*=0.0043)
